# Bilateral Serous Retinal Detachments Associated with Accelerated Hypertensive Choroidopathy

**DOI:** 10.4061/2010/964513

**Published:** 2010-09-01

**Authors:** Yoshio Hirano, Tsutomu Yasukawa, Yuichiro Ogura

**Affiliations:** Department of Ophthalmology & Visual Science, Nagoya City University Graduate School of Medical Sciences, 1-Kawasumi, Mizuho-cho, Mizuho-ku, Nagoya 467-8601, Japan

## Abstract

*Purpose*. We report a case of hypertensive choroidopathy with bilateral serous retinal detachments. *Patient*. A 50-year-old man underwent bilateral serous retinal detachments. Retinal arteriolar narrowing, vascular tortuosity, and arteriovenous nicking were identified in both eyes. The blood pressure was 206/125 mmHg. The patient was diagnosed with bilateral hypertensive choroidopathy and treated with oral antihypertensive treatment. *Results and discussion*. One month after antihypertensive treatment, the serous retinal detachments resolved and the visual acuity improved. A patient with those findings should be considered as having hypertensive choroidopathy and treated as soon as possible.

## 1. Introduction

Hypertensive disease, in which there is persistent pathologic elevation of arterial pressure and increased total peripheral resistance, is associated with vascular lesions in the brain, heart, kidneys, and eyes. Elevation of systemic blood pressure causes both focal and generalized retinal arteriolar constriction, presumably mediated by autoregulation. A prolonged duration of particularly high blood pressure can be associated with a breakdown of the inner blood-retinal barrier, with extravasation of plasma and red blood cells. Retinal hemorrhages, cottonwool spots, intraretinal lipid, and, in severe cases, the development of a macular star configuration of intraretinal lipid can be seen [1–3]. When the choroidal vessels are severely affected by elevated blood pressure, as in acute hypertension, fibrinoid necrosis of choroidal arterioles can cause occlusion of areas of choriocapillaris, with a subsequent breakdown of the outer blood-retinal barrier. In severe cases the optic nerve can be involved. Although the retinal vascular changes and optic neuropathy are well known, hypertensive choroidopathy usually does not receive as much attention. Hypertensive choroidopathy has been reported in toxemia of pregnancy, renal disease, pheochromocytoma, and malignant hypertension [4].

We report a case of hypertensive choroidopathy with bilateral serous retinal detachments.

## 2. Case Report

**Figure 1:**
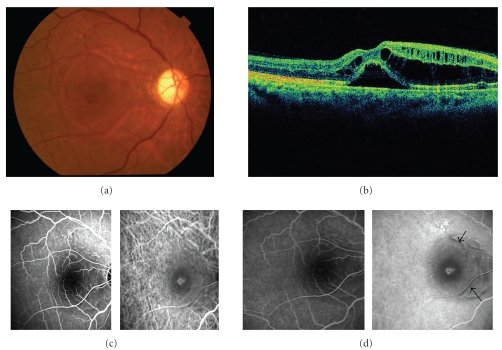
The right eye of a 50-year-old patient with severe hypertension at the first visit. (a) Fundus photograph shows serous retinal detachment. The optic disc is pale at the temporal side. (b) Optical coherence tomography (OCT) shows retinal detachment involving the fovea and cystic change of inner retina. (c) Early phase images of fluorescein angiography (FA) (*left*) and indocyanine green angiography (IA) (*right*). (d) Late phase images of FA (*left*) and IA (*right*). FA shows window defect associated with macular cystic change. Note that no active leakage is observed in the area with serous retinal detachment or cystoid edema. IA shows decreased perfusion of the choroid at the macula and window defect with the damaged RPE. Hypofluorescence (arrow) along the retinal artery are observed. These are unique findings.

**Figure 2:**
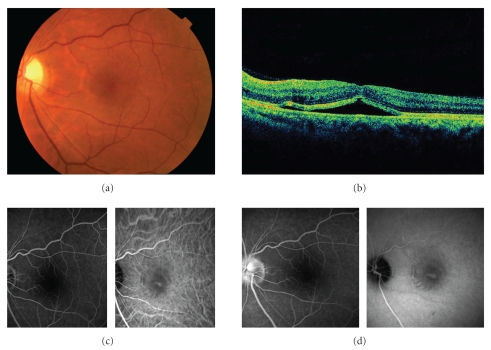
The left eye of the same patient at the first visit. (a) Fundus photograph shows serous retinal detachment. The optic disc is swelling. (b) OCT shows retinal detachment involving the fovea and cystic change of inner retina. (c) Early phase images of FA (*left*) and IA (*right*). (d) Late phase images of FA (*left*) and IA (*right*). Note that FA shows no active leakage corresponding to the area with serous retinal detachment and staining of the swollen optic disc. IA shows decreased perfusion of the choroid at the macula and window defect associated with damaged RPE.

**Figure 3:**
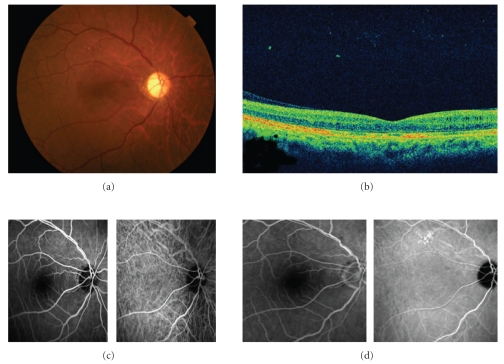
The right eye 1 month after treatment. (a) Fundus photograph shows the retina is reattached. (b) OCT shows that the retina and cystoids change are mostly improved. (c) Early phase images of FA (*left*) and IA (*right*). (d) Late phase images of FA (*left*) and IA (*right*). FA and IA show no remarkable findings. The hypofluorescence along the retinal artery disappeared.

**Figure 4:**
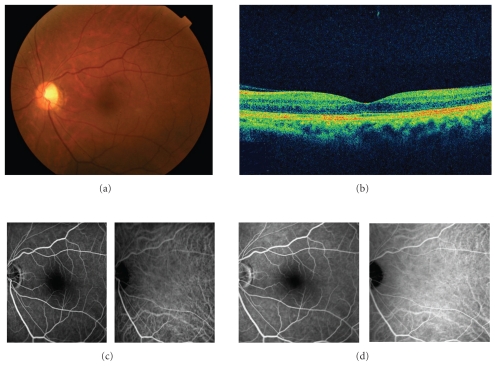
The left eye 1 month after treatment. (a) Fundus photograph shows the retina is reattached and optic disc is not swelling. (b) OCT shows that the retina is reattached. (c) Early phase images of FA (*left*) and IA (*right*). (d) Late phase images of FA (*left*) and IA (*right*). FA and IA show no remarkable findings.

A 50-year-old man presented with a painless loss of vision in his both eyes and dull, pressure-like frontal headache. The best-corrected visual acuity (BCVA) was 20/200 and 20/50 in the right and left eyes, respectively. He had a history of anterior ischemic optic neuropathy in the right eye 30 years ago. Slit-lamp examination showed serous retinal detachment in both eyes and optic disc swelling in the left eye (Figures 1 and 2). The optic disc in the right eye was pale. Aqueous cells and vitritis were not observed in both eyes. Retinal arteriolar narrowing, vascular tortuosity, and arteriovenous nicking were identified in both eyes. Fundus fluorescein angiography (FA) showed window defect associated with the macular cystoid change. Indocyanine green angiography (IA) showed decreased perfusion of the choroid at the macula in both eyes. His blood pressure (BP) was 206/125 mmHg. He had a history of hypertension 1 year ago, but had no medical treatment. The patient was diagnosed with bilateral hypertensive choroidopathy. He was treated with 40 mg/day of oral nifedipine. His BP was gradually improved. On posttreatment day 30, the serous retinal detachment disappeared in both eyes (Figures 3 and 4). The optic disc has become normal. His BCVA was recovered to 20/500 and 20/16 in the right and left eyes, respectively. The optic disc in the right eye remained pale due to anterior ischemic optic neuropathy 30 years ago.

## 3. Discussion

Severe systemic hypertension is associated with significant damage of end organs, such as eyes, heart, central nervous system, and kidneys. Choroidal lesions are less commonly recognized than retinal and optic nerve lesions [5].

The ophthalmic artery branch of the internal carotid artery supplies the optic nerve and retina, extraocular muscles, and eyelids. Branches of ophthalmic artery include the central retinal artery and short and long posterior ciliary arteries. The central retinal artery and vein supply the inner (anterior) two thirds of the retina. The choroid lies between the retinal pigment epithelium and sclera. The choroidal circulation receives blood primarily from the short and long posterior ciliary arteries and delivers blood to the retinal pigment epithelium, optic nerve, and outer (posterior) one third of the retina. The retinal photoreceptors and pigment epithelium are nourished by an inner layer of the choroid which is called the choriocapillaris.

Accelerated hypertension and/or ophthalmic/ciliary artery occlusion may result in choroidal ischemia. With complete ophthalmic artery occlusion, both the retinal and choroidal circulations are compromised. Compromise of ciliary arteries may occur without retinal artery involvement, leading to choroidal ischemia, which causes hypertensive choroidopathy. The RPE becomes necrotic; this may result in serous retinal detachment and/or localized pigment epithelial detachment. Some of these are attributed to a breakdown of the inner blood-retinal barrier with retinal endothelial cell decompensation. 

It is notable that no active leakage is observed in the area with serous retinal detachment in our patient. IA shows hypofluorescence along the retinal artery. Although it is unclear that this finding is cause or effect, it may play a role in the pathogenesis of this case.

The narrowed arterioles, vascular tortuosity, and arteriolovenous nicking in the retinal vessels were also observed. These findings are relatively common in longstanding hypertension. It seems that the patient had a history of longstanding chronic hypertension, and a sudden BP elevation triggered it off and formed this condition.

After antihypertension treatment, the serous retinal detachment disappeared, and his BCVA was recovered. In most cases with hypertensive choroidopathy, visual acuity returns to normal by controlling their BP. However, there is a case reporting that the visual acuity was not recovered in spite of being controlled the BP [6]. A patient with those findings should be considered as hypertensive choroidopathy and treated with antihypertension therapy as soon as possible.
